# Evaluation of two kinetic methods for serum amylase using a Cobas Bio centrifugal analyser

**DOI:** 10.1155/S146392468300036X

**Published:** 1983

**Authors:** Marion G. Anderson, Anne M. Kelly

**Affiliations:** Department of Biochemistry Hawkhead Hospital Hawkhead Road Paisley PA2 7BL UK


Journal of Automatic Chemistry, Volume 5, Number 3 (July–September 1983), pages 150–152

### Evaluation of two kinetic methods for serum amylase using a Cobas Bio centrifugal analyser

**Marion G. Anderson and Anne M. Kelly**

*Department of Biochemistry, Hawkhead Hospital, Hawkhead Road, Paisley PA2 7BL, UK*

### Introduction

End-point chromogenic methods for measuring serum amylase are still the most widely used in the UK, but there are theoretical grounds for preferring kinetic methods.

Recently, both Boehringer Mannheim and Beckman have introduced new kinetic colorimetric methods. Boehringer have based their method on the release of p-nitrophenol from p-nitrophenylmaltoheptaoside by amylase and added α-glucosidase [1]. The Beckman method employs maltotetraose as substrate and the auxiliary enzymes maltose phosphorylase, β-phosphoglucomutase and glucose-6-phosphate dehydrogenase, see Pierre *et al.* [2].

This paper describes the assessment of both methods using a Cobas Bio centrifugal analyser and compares them with the Phadebas method reported by Ceska *et al.* [3].

### Materials and methods

The reagents in Boehringer kit No. 568589 and Beckman kit No. 682367 were reconstituted according to the supplier's instructions and stored at 4°C. The parameters for the Boehringer method for the Cobas Bio were:

BIOCHEMISTRY HAWKHEAD HOSPITAL

TEST NR 19 COBAS BIO

PARAMETER LISTING

| | | |
|---|---|---|
| 1 | UNITS | U/L |
| 2 | CALCULATION FACTOR | 14152 |
| 3 | STANDARD 1 CONC | 0 |
| 4 | STANDARD 2 CONC | 0 |
| 5 | STANDARD 3 CONC | 0 |
| 6 | LIMIT | 1 |
| 7 | TEMPERATURE [DEG. C] | 37·0 |
| 8 | TYPE OF ANALYSIS | 3 |
| 9 | WAVELENGTH [NM] | 405 |
| 10 | SAMPLE VOLUME [UL] | 05 |
| 11 | DILUENT VOLUME [UL] | 05 |
| 12 | REAGENT VOLUME [UL] | 200 |
| 13 | INCUBATION TIME [SEC] | 10 |
| 14 | START REAGENT VOLUME [UL] | 20 |
| 15 | TIME OF FIRST READING [SEC] | 120·0 |
| 16 | TIME INTERVAL [SEC] | 10 |
| 17 | NUMBER OF READINGS | 21 |
| 18 | BLANKING MODE | 1 |
| 19 | PRINTOUT MODE | 1 |

Contrary to the manufacturer's instructions the reagents were not combined. This allowed longer stability of the reagents, and if stored at 4°C in Cobas Bio reagent boats they only required to be routinely changed at weekly intervals. α-glucosidase was placed in the main reagent well and substrate in the start reagent well.

A preliminary limit of 1·0 Au was set. This corresponds to an amylase activity of approximately 10 500 IU/l (by the Phadebas method) and was the highest activity obtained. Substrate exhaustion did not occur during analysis at this activity and zero-order kinetics were maintained throughout. A 120 s incubation was found to be necessary for sera with normal amylase activities.

The parameters for the Beckman method on the Cobas Bio were:

BIOCHEMISTRY HAWKHEAD HOSPITAL

TEST NR 27 COBIAS BIO

PARAMETER LISTING

| | | |
|---|---|---|
| 1 | UNITS | U/L |
| 2 | CALCULATION FACTOR | 8039 |
| 3 | STANDARD 1 CONC | 0 |
| 4 | STANDARD 2 CONC | 0 |
| 5 | STANDARD 3 CONC | 0 |
| 6 | LIMIT | 0 |
| 7 | TEMPERATURE [DEG. C] | 37·0 |
| 8 | TYPE OF ANALYSIS | 2 |
| 9 | WAVELENGTH [NM] | 340 |
| 10 | SAMPLE VOLUME [UL] | 05 |
| 11 | DILUENT VOLUME [UL] | 05 |
| 12 | REAGENT VOLUME [UL] | 100 |
| 13 | INCUBATION TIME [SEC] | 0 |
| 14 | START REAGENT VOLUME [UL] | 0 |
| 15 | TIME OF FIRST READING [SEC] | 300·0 |
| 16 | TIME INTERVAL [SEC] | 10 |
| 17 | NUMBER OF READINGS | 20 |
| 18 | BLANKING MODE | 1 |
| 19 | PRINTOUT MODE | 1 |

The reagents were used as directed by Beckman. The 300 s incubation was found to be necessary for the reaction to become linear. Substrate exhaustion did not occur at an activity of 8000 IU/l (Phadebas method) using these parameters.

Phadebas amylase test tablets were obtained from Pharmacia Diagnostics AB of Uppsala, Sweden, and the assay performed according to instructions. All reactions were carried out at 37°C. Sera with amylase activities ranging from 71 IU/l to 10 500 IU/l (determined by the Phadebas method) were obtained from hospital patients and from local general practitioners' patients.

### Results

### *Linearity*

A pathological serum with amylase activity of 10 500 IU/l was diluted with saline to give various activities down to 656 IU/l, and the samples were assayed by the Boehringer method. The method was shown to be linear over this range. Similarly, the Beckman method was found to be linear up to an activity of at least 8100 IU/l.

### *Precision*

Table 1 shows within-batch and between-batch CVs over a wide range of amylase activities. Within-batch CVs were determined on 10 replicates and between-batch CVs on 10 consecutive assays.

*Table 1. Precision studies.*

| (1) | Boehringer amylase activity (IU/l) Mean | SD | CV(%) |
|---|---|---|---|
| Within-batch | 72 | 3·6 | 5·0 |
| | 325 | 19·7 | 6·1 |
| $N=10$ | 597 | 14·2 | 2·4 |
| | 4750 | 57·2 | 1·2 |
| Between-batch | 76 | 5·1 | 6·7 |
| | 154 | 8·4 | 5·5 |
| $N=10$ | 310 | 12·3 | 4·0 |
| | 603 | 24·8 | 4·1 |
| | 1870 | 98·0 | 5·2 |
| **(2)** | **Beckman amylase activity (IU/l) Mean** | **SD** | **CV(%)** |
| Within-batch | 21 | 1·0 | 4·7 |
| | 44 | 1·2 | 2·7 |
| $N=10$ | 299 | 7·2 | 2·4 |
| | 715 | 6·3 | 0·9 |
| Between-batch | 77 | 2·3 | 3·0 |
| | 144 | 6·6 | 4·6 |
| $N=10$ | 397 | 12·2 | 3·1 |
| | 490 | 16·8 | 3·4 |

### *Comparison with Phadebas method*

Amylase activities ranging from 90 IU/l to 10 500 IU/l were assayed in 167 samples of serum by the Boehringer method and by the Phadebas method. Figure 1 shows part of the correlation graph. The Phadebas method gave consistently higher results, the regression equation being $y=1{\cdot}81x+34$ and the correlation coefficient 0·986. Similarly, the Beckman and Phadebas methods were compared for 133 sera over the range 71 IU/l to 8100 IU/l. In this case, a biphasic correlation was found. Sera which had normal amylase activities, i.e. <300 IU/l by the Phadebas method, gave a regression equation of $y=2{\cdot}4x+30$ with a correlation coefficient of 0·866 (see figure 2[*a*]). Sera with elevated amylase activities (i.e. >300 IU/l) gave an equation of $y=5{\cdot}5x-208$ with $r$ being 0·980 (see figure 2[*b*]). When the Boehringer comparison was broken down in a similar manner this phenomenon was less obvious. Sera with activities greater than 300 IU/l gave a regression equation of $y=1{\cdot}85x+69$, and those with activities <300 IU/l gave the equation $y=1{\cdot}48x+57$.

### *Reference ranges*

Reference ranges were determined using 117 sera (Boehringer) and 109 sera (Beckman), which had normal amylase activities by the Phadebas method (less than 300 IU/l). The mean activity for the Boehringer method was found in this study to be 97 IU/l, giving a reference range of 32–162 IU/l (mean ± 2SD); this compares with the range of less than 180 IU/l given in the Boehringer literature for the method. The Beckman mean was 56 IU/ml giving a reference range of 14–98 IU/l (mean ± 2SD); Beckman quote a range of 20–110 IU/l at 37°C.

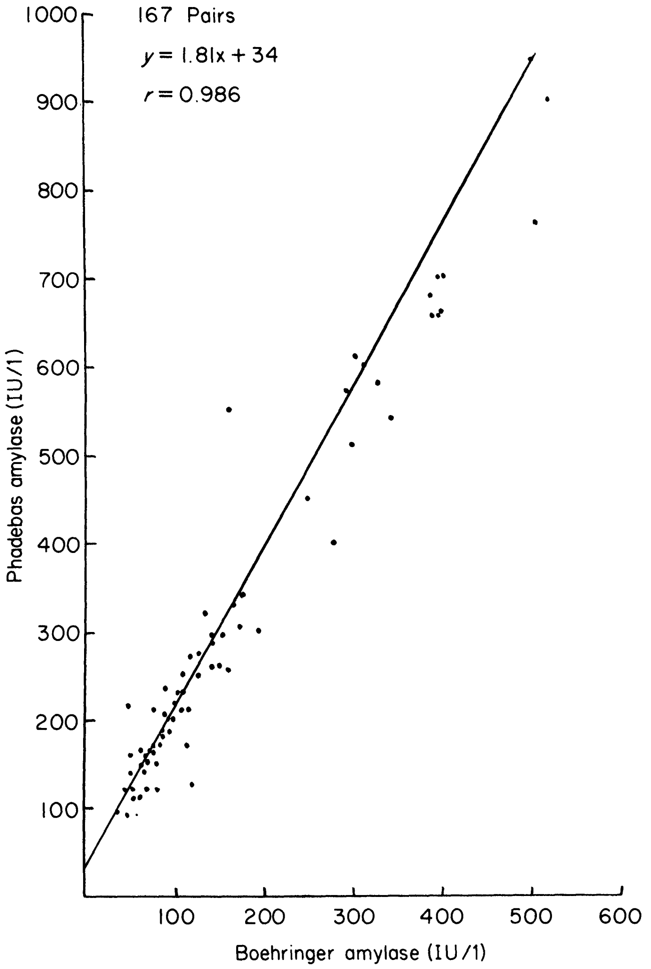

*Figure 1. Comparison of the Phadebas and Boehringer methods.*

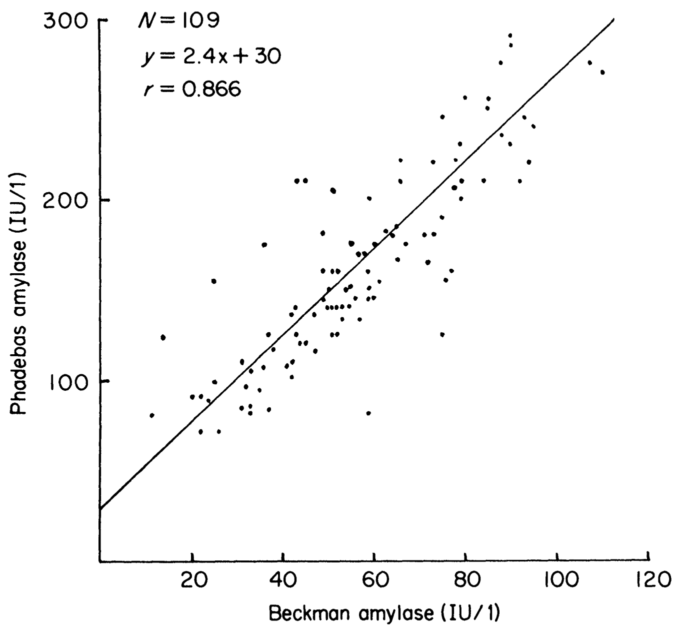

*Figure 2(a). Comparison of the Phadebas and Beckman methods for normal amylase activities.*

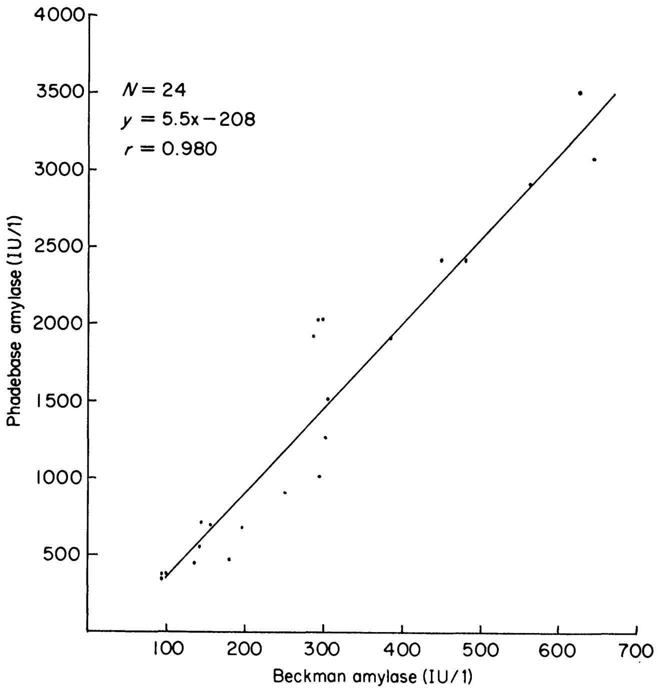

*Figure 2(b). Comparison of the Phadebas and Beckman methods for elevated amylase activities.*

### Discussion

In this investigation both kinetic methods have proved to be precise, convenient and easily adapted to the Cobas Bio centrifugal analyser. Their main advantages over the Phadebas method are:

(1) Substrate exhaustion does not occur, even at very high amylase activities (i.e. in the region of 8000 to 10 000 IU/l). Using the Phadebas method these sera would require dilution and repetition.

(2) Both methods are precise, with between-batch CVs of 3–6%; the Beckman method is marginally better.

(3) In the Boehringer method, by storing the reagents separately in the Cobas Bio boats, they remain stable for up to two weeks. This allows more assays to be performed from each vial of reagent. Once reconstituted, the Beckman reagent is only stable for two days at 4°C. Storage of the reagents in the boats also speeds up emergency analyses performed outside normal working times.

(4) Because the reagent volumes are scaled down considerably by using the Cobas Bio, both methods compare very favourably in terms of cost with the Phadebas method. An amylase estimation using the Phadebas tablets to include blank, test serum and quality-control serum costs (May 1983) approximately £0·56. Using the kit methods in a similar 'one-off' mode the cost is about £0·24 and £0·23 for the Boehringer and Beckman methods respectively.

A biphasic type of correlation was found when comparing the Phadebas and Beckman. The phenomenon was noted to a much lesser degree when comparing the Phadebas and Boehringer methods. An explanation could be that amylase in normal serum appears to consist mainly of isoenzymes from the pancreas and salivary glands [4], with salivary amylase predominating [5], whereas in pathological sera the amylase is almost entirely pancreatic in origin. The pancreatic isoenzyme may react at a different rate from the salivary form with the different substrates used in the kits. From a practical point of view, provided that a reference range has been determined for the selected method, such differences in isoenzyme reactivity do not affect interpretation of results and the normal and the abnormal can be distinguished.

### References

1. Rauscher, E. *et al.*, *Proceedings of the Third International Congress on Clinical Enzymology* (Salzburg, 1981).
2. Pierre, K. J., Tung, K. K. and Nadj, H., *Clinical Chemistry*, **22** (1976), 1219.
3. Ceska, M., Birath, K. and Brown, B., *Clinica Chimica Acta*, **22** (1969), 437.
4. Berk, J. E., Searcy, R. L., Hayashi, S. and Ujihara, I., *Journal of the American Medical Association*, **192** (1965), 389.
5. Meites, S. and Rogols, S., *Clinical Chemistry*, **14** (1968), 1176.

### IUPAC 1985

*The 30th International Congress of Pure and Applied Chemistry*

The 30th IUPAC Congress will be held from Monday 9 to Friday 13 September 1985 in Manchester, UK. The opening ceremony and the inaugural plenary lecture will take place on Monday morning; the meeting will end at lunchtime on Friday. A full programme of evening and daytime social events is being planned.

The Royal Society of Chemistry is responsible for the detailed organization of the Congress.

The scientific programme will embrace the following Divisions and topics, and prominent chemists from all over the world are being invited to present lectures. The symposia within the individual sections will take place both concurrently and sequentially. It is planned to include free-offer contributions, particularly as posters. Details regarding submission of papers will be notified in a circular, which will be available in March 1984.

*Section 1: Analytical (Analytical Division)*
New instrumental methods
Advances in automatic methods
Environmental analyses
Biotechnology–analytical applications.

*Section 2: Education (Education Division)*
Chemical education research
Laboratory work: assumptions and realizations
Continuing education in chemistry
Changing requirements for manpower skills (jointly with Industrial Division).

*Section 3: Industrial (Industrial Division)—Chemical Industry—Year 2001*

*Section 4: Inorganic (Dalton Division)*

*Section 5: Organic (Perkin Division)—Organic Chemistry as a Life Science*

*Section 6: Organic (Perkin Division)/Historical*

*Section 7: Physical (Faraday Division)—Advances in Physical and Theoretical Chemistry*

*Requests to be added to the mailing list for circulars to the Royal Society of Chemistry, Burlington House, London W1V 0BN.*

